# The Importance of Developmental Assets to Mental Health in Norwegian Youth

**DOI:** 10.3389/fpsyg.2021.687537

**Published:** 2021-07-14

**Authors:** Nora Wiium, Marianne Beck, Laura Ferrer-Wreder

**Affiliations:** ^1^Department of Psychosocial Science, University of Bergen, Bergen, Norway; ^2^Department of Educational Psychological Services, Bergen, Norway; ^3^Department of Psychology, Stockholm University, Stockholm, Sweden

**Keywords:** developmental assets, prolonged sadness, suicide attempt, youth, Norway

## Abstract

In the present study, we examined the importance of developmental assets to prolonged sadness (i.e., being sad most of the time or all the time for no reason in the last month) and suicide attempt. Cross–sectional data on items measuring developmental assets as well as prolonged sadness and suicide attempt were collected from high school students in Norway (*N* = 591, 55% girls). The findings from independent *t*–tests indicated that youth with poor mental health reported less developmental assets relative to their peers who did not report such problems. In logistic regression, asset categories, such as Positive identity and Personal assets, were significantly associated with poor mental health (especially prolonged sadness) after adjusting for other asset categories and demographic factors, such as age, sex, and parents' educational background. The influence of Empowerment and Family assets, which was significant when only the assets were assessed, was no longer significant when demographic variables were also considered. While more research on factors that can promote youth mental health is needed, our findings suggest that policies and programmes that ensure that youth have access to the necessary developmental resources and opportunities may also be empowering youth, enhancing their mental health, and consequently, facilitating their active involvement in their community.

## Introduction

The World Health Organization defined mental health as “a state of well–being in which the individual realizes his or her own abilities, can cope with the normal stresses of life, can work productively and fruitfully, and is able to make a contribution to his or her community” (World Health Organization, 2004, p. 59). Accordingly, the mental health of individuals at different stages of life is important to how they think, feel, act, and interact with others. While having good mental health will mean being able to participate in self and societal development, the opposite may also be the case. Individuals with poor mental health may not only be more likely to suffer from social isolation and alienation; they may also be excluded from economic participation (Hall et al., 2019). Thus, there is a clear need to bolster protection at the individual and contextual levels in order to diminish the chances and effects of poor mental health. The present study considers such protective factors at the individual and contextual levels.

Globally, at least 10% of the world's population suffers from mental, neurological and substance use disorders, while among children and adolescents, about 20% are believed to suffer from some form of mental disorder (Jamison et al., 2016). Suicide is an indicator of poor mental health, common in adolescents and it occurs when an individual purposefully kills him or herself. Suicide is the third leading cause of death in 15–19–year–olds (World Health Organization, 2021). In a study involving 82 low to high income countries, Biswas et al. (2020) found that 14% of adolescents between ages 12 and 17 years reported suicidal ideation during a 12–month period. Persistent or prolonged sadness (American Psychiatric Association, 2013) has also been used as a proxy of poor mental health in earlier studies (Brosnahan et al., 2004; Tebeka et al., 2018; Garcia et al., 2019).

Due to the immense personal, social, and economic implications of poor mental health, urgent actions are needed to address the condition. Within the Positive Youth Development (PYD) perspective, the probability of experiencing poor mental health can be reduced if young people are provided with the necessary social resources and empowerment opportunities to learn, lead, and grow in different contexts, such as home, school and community (Benson et al., 2011). In addition, these resources and opportunities, collectively called developmental assets, can be protective against poor mental health that is common in young people. It is this theoretically hypothesized protective effect of the assets that we focus on in the present study, by investigating the associations of several developmental assets, for example, support from school and home, as well as personal assets like social competence on poor mental health indicators, such as prolonged sadness and suicide attempt in Norwegian youth.

### Developmental Assets and The Positive Youth Development Perspective

Within the framework of Positive Youth Development, developmental assets are the building blocks of positive development (Benson, 2007). The presence of these assets is thought to ensure a dynamic bidirectional relationship between an active, engaged, and competent youth and ecologies that are receptive, supportive, and nurturing, thereby leading to the so–called adaptive developmental regulations, where both youth and their context benefit (Lerner et al., 2015). Specifically, it is posited that young people who are thriving are also more likely to contribute to their own development as well as to the society they are part of. The theoretical assumption is also that thriving youth or youth with more resources will report fewer problems or risk behaviors (Lerner et al., 2017).

In 1990, Peter Benson and the Search Institute in Minneapolis, U.S.A. launched 40 developmental assets, 20 internal and 20 external assets [i.e., youth strengths and contextual assets, respectively; (Benson, 1990, 2007)]. These assets were hypothesized to be what young people need to thrive or develop in a healthy way. Internal assets have four sub–categories: Commitment to learning (e.g., achievement motivation, and school engagement), Positive values (e.g., integrity and responsibility), Social competencies (e.g., planning and decision–making, and resistance skills) and Positive identity (e.g., self–esteem and sense of purpose). Likewise, there are four sub–categories of external assets: Support (e.g., family support and caring school climate), Empowerment (e.g., how the community values youth and community's perception of youth as resources), Boundaries & Expectations (e.g., family boundaries and significant others' expectations of young people), and Constructive use of time (e.g., in creative activities and youth programmes) (Benson, 2007).

The eight asset categories together constitute developmental assets in five different contexts: Personal, Social, Family, School, and Community. Within Bronfenbrenner's ecological theory (Bronfenbrenner, 2005), the assets will be located mostly in the micro– and meso–system, where youth development (and in the case of the current study, poor mental health) would be a function of the individual's interaction with immediate contexts, including the family, school, and local community, as well as the effective collaboration existing between the different immediate contexts. Other systems in the ecological theory, such as the exo– and the macro–systems refer to conditions in the more distal youth and cultural contexts (e.g., policies that define what are the social priority areas in organizations/contexts as well as resource allocation at school and larger community).

The theoretical assumption of the Asset model is that a vertical pile–up (i.e., experiencing more assets in an asset category) and horizontal stacking (i.e., experiencing assets across different asset categories) of the assets will promote positive youth outcomes and prevent negative development. Although the assets were determined based on youth samples living in the U.S., growing research has confirmed their presence in several non–U.S. samples as well as their psychometric properties across different ethnic groups and countries (Scales, 2011; Scales et al., 2017; Wiium et al., 2018). Empirically, the facilitating role of the developmental assets on positive youth outcomes and their protection against different types of problems have also been observed in youth samples in Africa (Adams et al., 2018), Europe (Issa et al., 2020) and Latin America (Manrique–Millones et al., 2021).

### Empirical Evidence of the Protective Role of Developmental Assets

As the current study focuses on the possibility of a protective role for developmental assets, we now describe several relevant empirical studies within this area of inquiry. We consider both studies that have examined developmental assets using Peter Benson and the Search Institute's Developmental Assets Framework as well as those that have considered other PYD indicators, in relation to their associations with poor mental health indicators.

In a study of 451 college students living in the Midwest United States, Pashak et al. (2018) observed inverse and significant associations between a global measure of developmental assets and several risk and mental health indicators, including tobacco, alcohol, and other drug use as well as depression and suicidal thoughts. Moreover, these significant associations were also observed with global scores of internal (e.g., social competence) and external (e.g., support) assets. Thus, having more internal assets, external assets or assets in general were protective against risk behaviors and mental health problems. As well, Lenzi et al. (2015) studied the relations between protective assets, in terms of quantity, variety, and configuration (i.e., alignment between quantity and variety) and risk behaviors (tobacco and alcohol use) along with emotional problems (depressive feelings and suicidal thoughts). With a sample of over 12,000 U.S. high school students, Lenzi and colleagues observed that having a high quantity of assets, as well as a variety of assets was associated with a lower probability of experiencing risk behaviors and emotional problems. Furthermore, when configurations of quantity and variety of assets were considered, a higher number of assets together with a greater variety of asset domains were associated with reduced behavioral and emotional problems. The studies described by Pashak et al. (2018) and Lenzi et al. (2015) highlight evidence in support of the protective effects of youth assets, however the assets were examined in these studies as composite factors, thus disguising the distinct role of particular assets.

Considering the possibility of the discrete role of assets, in a study of 1,111 ethnically diverse U.S. youth aged 12–17 years old, Lensch et al. (2018) assessed the prospective associations between sixteen youth assets and suicide ideation, as well as the cumulative effects of the assets at the individual–, family– and community–levels on suicide ideation using data collected from four waves of the Youth Asset Study. Assessing the individual effects of the assets, Lensch et al. (2018) found prospective and protective associations between several individual–level assets (e.g., responsible choices, general self–confidence and good health practices), family–level assets (e.g., family communication, parental monitoring and relationship with mother) and community–level assets (e.g., use of time for groups and sports, school connectedness and community involvement) and suicidal ideation. Moreover, in their assessment of the cumulative effects of the assets, the authors observed a graded protective association within each asset domain, where an increase in the number of assets reported was associated with a decrease in the odds of suicidal ideation.

Furthermore, other indicators of positive youth development, such as the Chinese Positive Youth Development Scale (reflecting cognitive–behavioral competence, prosocial attributes, general PYD qualities like bonding and resilience, as well as positive identity) was found to be protective against problem behaviors and poor mental health (Zhou et al., 2020). Thus, skills and resources may have a buffering role in regards to protecting young people against negative development.

### The Norwegian Context

Although there are several youth services within the school system and at the municipality level to support the mental health of Norwegian youth, the available statistics still show increasing prevalence of poor mental health, especially among adolescent girls (Norwegian Institute of Public Health, 2019). Suicide rates in Norway have remained steady over a 20–year period, between 1995 and 2015 (Ekeberg and Hem, 2019). While suicide among children and young adolescents is rare, among 15–24–year–olds, about 15 suicides per 1,00,000 inhabitants per year among males and 6 among females were registered between 2014 and 2018. At the same time, the 2019 report by the Norwegian Institute of Public Health on quality of life and mental health among children and adolescents indicated that over 90% of them reported they were satisfied or very satisfied with their life. In fact, compared to other OECD countries, Norway has usually been rated as one of the countries with a higher level of life satisfaction (Martela et al., 2020). Traditionally, mental health services have addressed poor mental health from the deficit–based approach. Strengths–based approaches that may not only protect against mental health problems but enhance mental wellbeing and address the holistic development of young people as well, would be an important addition to youth mental health services.

The focus of the Norwegian national youth policy from 2002 on strategic areas, such as comprehensive preventative work, education and schools, efforts intended for leisure and community, support of children and adolescents with serious behavioral problems, follow–up of young offenders and criminal youth gangs as well as knowledge and research (Youth Policy: Norway, 2014), indicates that Norway has what Benson (2007) refers to as asset–building community and asset–building society for positive youth development. Accordingly, a sustained implementation of these national strategies that are traditionally “grassroots” and “decentralized” to local municipalities (Bergan, 2017), can ensure that behaviors and programmes (i.e., asset–building community) as well as norms and policies that support them (i.e., asset–building society) are in place to provide Norwegian youth with the needed developmental resources; resources that can protect against or enhance mental well–being. Moreover, efforts made to ensure gender equality regarding access to education and employment (Equality, 2014) can have both promotion and protective effects. Indeed, the Global Gender Gap index that reflects females' prospects in four areas (economic participation and opportunity, educational attainment, health and survival, and political empowerment) in relation to men, ranks Norway as number two out of 153 countries with an overall score of 0.842 (World Economic Forum, 2020).

### The Present Study

The aim of the present study is to explore the role of Benson (2007) developmental asset categories on poor mental health indicators (prolonged sadness and suicide attempt) in Norwegian youth. The use of strengths–based approaches in Norwegian youth studies is scarce. Findings of the few studies that have been carried out indicate that developmental assets, for example, Commitment to learning, Support and Positive identity were significant predictors of positive youth outcomes (e.g., academic achievement; Beck and Wiium, 2019). With the use of the PYD perspective in the present study to examine the protective role of developmental assets against poor mental health in Norwegian youth, we are not only providing some insights into how positive youth development strategies can be studied within a well–established framework, but we are also presenting a context where positive development can be advanced in youth programme and policies in Norway. In the analysis, we also examine the developmental assets in the five environments (Personal, Social, Family, School, and Community) to determine the relative relevance of each category.

We anticipate negative associations between the asset categories and poor mental health indicators where lower scores on the assets will be associated with a higher likelihood of prolonged sadness and suicide attempt. Similarly, we hypothesize that the five environmental asset categories will be negatively associated with poor mental health. Demographic factors, such as age, gender and parents' educational background have been found to have some implications for the report of the assets (Drescher et al., 2012; Wiium et al., 2018), and thus, are adjusted for in our study.

## Materials and Methods

### Participants

As part of a cross–national research project (Wiium and Dimitrova, 2019), the current study uses cross–sectional data that were collected from 591 students attending a public high school that had recently undergone a structural change, the combination of four schools into one regional school. The age range of participants was 15–19 years (*M*_*age*_ = 16.70, *SD* = 0.90), with gender distribution as 55% girls. More than half of the participants reported that their parents' highest education was post–secondary (college or university); about 56% reported post–secondary education as their father's highest level of education while the corresponding percentage for participants' mother was 67% (Table 1).

**Table 1:** Descriptive statistics of study variables.

	**Total sample *N* = 591**
Age, Mean (*SD*)	16.70 (0.90)
Gender, % Females	55
Father's education, % post–secondary	56
Mother's education, % post–secondary	67
Prolonged sadness %	18
Suicide attempt %	6
The eight asset categories, α	
Commitment to learning	0.84
Positive Values	0.73
Social competencies	0.80
Positive identity	0.86
Support	0.81
Empowerment	0.77
Boundaries & Expectations	0.80
Constructive use of time	0.44
The five environmental asset categories, α	
Personal	0.83
Social	0.83
Family	0.86
School	0.85
Community	0.65

*SD—Standard Deviation; α–Cronbach's alpha*.

### Measures

*Developmental assets* (Search Institute, 2016). Participants indicated the extent to which they had experienced Benson (2007) developmental assets: the four internal asset categories (i.e., Commitment to learning—seven items, Positive values—seven items, Social competencies—seven items, and Positive identity—four items) and the four external asset categories (i.e., Support—seven items, Empowerment—six items, Boundaries & Expectations—nine items, and Constructive use of time—four items). Sample items for internal assets were related to how participants cared about school, told the truth even when it is not easy, were sensitive to the needs and feeling of others and whether they felt they had control of their life and future, respectively. For external assets, sample items were related to whether participants had support from adults other than their parents, whether they felt valued and appreciated by others, if they had a family that knew where they were and what they were doing and whether they were involved in a sport, club, or other group activity, respectively. Responses were rated on a 4–point Likert scale: (1) not at all or rarely, (2) somewhat or sometimes, (3) very or often, and (4) extremely or almost always. For the asset categories in the five environments (Personal−11 items, Social−12 items, Family—nine items, School−10 items, and Community—nine items), sample items were related to whether participants told other people what they believed in, whether they expressed their feelings in proper ways, whether they had a family that provided them with clear rules, whether they did their homework and if they lived in a safe neighborhood. Cronbach's alpha for the eight developmental asset categories and asset categories in the five environments ranged from 0.73 to 0.86, except Constructive use of time that had a Cronbach's alpha of 0.44 (Table 1). The Cronbach's alpha values found in this study are like those found in earlier studies (e.g., alpha values ranging from 0.60s to 80s; Scales et al., 2000). In our analysis, we considered all asset categories.

*Poor mental health indicators* (Search Institute, 2016). Two indicators were assessed: prolonged sadness and suicide attempt. For prolonged sadness, participants were asked to indicate whether they have been sad most of the time or all the time for no reason in the last month, and for suicide attempt, participants indicated whether they had tried to commit suicide one or more times. In both cases, the response options were (1) No and (2) Yes. Due to the sensitive nature of the item on suicide attempt, participants were asked to seek help from the school health services if any of our questions evoked concern, unrest or otherwise. Similar single item measures of poor mental health indicators have been used by previous studies (Garcia et al., 2019; Toomey et al., 2019). Nonetheless, we address our use of single item measures in the assessment of poor mental health as a limitation in the discussion section.

*Demographics*. Participants were asked to provide information on demographic variables, such as age, gender (i.e., boy or girl) and the highest educational level of their father and mother (i.e., no education, primary school, high school, technical or vocational school and University education). These variables were treated as control variables as they have been found to have some implication for the experience of developmental assets (Wiium et al., 2018).

### Procedure

The present study was approved by the Regional Committee for Medical and Health Research Ethics in Norway (2014/1645). Students at all three levels of the high school were invited to participate in the study. Data were collected with a response rate of 70%. Prior to that, the school and participants gave their informed consent, which happened after they had been informed about the study's goals and procedure. Data collection was conducted during school hours and lasted for about 40 min. Students had access to the questionnaire over the school's internal web system. Semantix Translations Norway AS, a company that specializes in interpreting and translation services, translated the questionnaire from English to Norwegian, ensuring preservation of meaning through double–checking and the use of translation experts in the relevant field of research.

### Data Analysis

Descriptive analyses together with independent *t*–test analyses were conducted using IBM SPSS Statistics for Windows, version 25, while all other analyses were performed using Mplus version 8 (Muthén and Muthén, 1998–2017). Most participants (97%) had missing on only three cases or less. All analyses in Mplus were conducted using the Maximum likelihood estimation, an estimation method that was used to handle missing cases. With this method, a likelihood function for each case is estimated based on the variables present in the dataset such that all the available data are used. The developmental assets were the independent variables, while the poor mental health indicators were the dependent or outcome variables.

For the different steps of the data analysis, descriptive analysis was first performed on demographic variables to investigate their pattern of distribution, while reliability analyses were undertaken on the developmental asset categories to determine Cronbach's alphas. Prior to the assessment of the associations between the developmental assets and mental health indicators, measurement invariance (MI) testing (i.e., configural invariance, metric invariance and scalar invariance) across the two response alternatives (no or yes) for each of the indicators (prolonged sadness and suicide attempt) was conducted. Such MI procedures allow for meaningful group comparisons of the asset categories in terms of factor structure, associations and factor means, respectively (Lee, 2018). MI was carried out using a set of Multigroup Confirmatory Factor Analyses (MGCFA) on the items measuring the eight developmental asset categories and the five environmental asset categories. After MI, independent *t*–test analyses were performed as the next step of the analysis to ascertain differences in the report of the assets across the two response alternatives that indicated whether there was a mental health problem or not. Thus, this analysis was to examine whether participants who did not report mental health problems were scoring higher on the assets than their peers who did. The independent *t*–test analyses were followed by a correlation analysis that was conducted to assess the degree to which the developmental assets and poor mental health were related. Finally, logistic regression models were analyzed to explore the importance of the asset categories in relation to each of the two mental health indicators in model 1 and controlling for the demographic variables in model 2. The logistic regression analysis was first carried out to assess the importance of the eight developmental asset categories and then of the five environmental asset categories.

To conduct the analyses, composite variables that reflected the number of assets reported for each of the asset categories (i.e., the eight developmental assets and the five environmental asset categories) were created. The four original response alternatives on each asset item were recoded, so that (1) “Never or rare” and (2) “Sometimes” were recoded as asset not present, and (3) “Often” and (4) “Almost always or Very often” recoded as asset present. This was done to determine the number of assets that were being reported and to assess their cumulative effects.

## Results

### Measurement Invariance of the Asset Categories

To evaluate model fit in the measurement models, chi–square tests and fit indices, such as the Tucker Lewis Index (TLI; acceptable > 0.90), the Root Mean Square Error of Approximation (RMSEA; acceptable below 0.08), and Comparative Fit Index (CFI; acceptable > 0.90) (Hu and Bentler, 1999; Byrne, 2008) were used. Results from the series of Multigroup Confirmatory Factor Analyses (MGCFA) conducted for the asset categories indicated that for the eight developmental asset categories, partial scalar invariance was established across the two statuses (response alternatives) of prolonged sadness (χ^2^(48, *N* = 548) = 97.10, *p* < 0.001, RMSEA = 0.061, CFI = 0.957, TFI = 0.950), while scalar invariance was established for suicide attempt (χ^2^(50, *N* = 548) = 135.48, *p* < 0.001, RMSEA = 0.079, CFI = 0.937, TFI = 0.929). For the five environmental asset categories, scalar invariance was established for both prolonged sadness and suicide attempt (χ^2^(16, *N* = 548) = 33.04, *p* < 0.001, *RMSEA* = 0.062, *CFI* = 0.980, *TFI* = 0.975) and (χ^2^(16, *N* = 548) = 42.27, *p* < 0.001, *RMSEA* = 0.077, *CFI* = 0.974, *TFI* = 0.967), respectively (Table 2).

**Table 2:** Measurement invariance models for developmental assets by mental health indicators.

**Model**	**Model fit indices**							
	***χ^2^ (df)***	***RMSEA***	***90% CI RMSEA***	***CFI/TLI***	***χ^2^ (df)***	***RMSEA***	***90% CI RMSEA***	***CFI/TLI***
The Eight Developmental Asset Categories								
	Prolonged sadness					Suicide attempt		
Configural invariance	85.22 (38)	0.067	0.048–0.087	0.959/0.940	108.36 (38)	0.082	0.064–0.101	0.948/0.923
Metric invariance	91.73 (44)	0.063	0.045–0.081	0.959/0.947	127.86 (44)	0.083	0.067–0.100	0.938/0.921
Scalar invariance	162.80 (50)	0.091	0.075–0.106	0.902/0.891	*135.48 (50)*	*0.079*	*0.063–0.095*	*0.937/0.929*
Partial scalar invariance	*97.10 (48)*	*0.061*	*0.043–0.079*	*0.957/0.950*				
The Five Environmental Asset Categories								
	Prolonged sadness					Suicide attempt		
Configural invariance	14.38 (8)	0.054	0.000–0.098	0.993/0.981	21.04 (8)	0.077	0.038–0.118	0.987/0.969
Metric invariance	17.84 (12)	0.042	0.000–0.080	0.993/0.989	36.86 (12)	0.087	0.056–0.120	0.975/0.958
Scalar invariance	*33.04 (16)*	*0.062*	*0.031–0.092*	*0.980/0.975*	*42.27 (16)*	*0.077*	*0.049–0.106*	*0.974/0.967*

*χ^2^, Chi–Square; df, degrees of freedom; CFI, Comparative Fit Index; TLI, Tucker Lewis Index; RMSEA, Root Mean Square Error of Approximation; CI, Confidence Interval; Configural, same number of factors and factor loading pattern across groups; Metric, equality of the factor loadings; Scalar, equality of the factor loadings and intercepts*.

### Associations Between the Asset Categories and Poor Mental Health Indicators

Results from correlation analyses not presented in tables revealed weak to strong correlations among the eight developmental asset categories (0.22 to 0.62) and medium to strong correlations among the five environmental asset categories (0.42 to 0.63). In addition, a Spearman's rank correlation between prolonged sadness and suicide attempt was weak, but significant (*r*_*s*_ = 0.20, *p* < 0.01). There was no significant correlation between gender and suicide attempt, while a weak, but significant correlation was observed between gender and prolonged sadness, with females scoring higher relative to males (*r*_*s*_ = 0.11, *p* < 0.05). From the results of the independent *t*–tests that were used to investigate the experience of the assets among participants (18%) who indicated that they had felt sad most or all the time without cause in the last month (i.e., prolonged sadness) and those who did not (82%), the former was observed to report significantly lower levels of assets on all eight categories of developmental assets compared to the latter, except for the assets relating to Constructive use of time (See Table 3). Similarly, the results of the independent *t*–tests denoted that participants who indicated that they had attempted suicide before (6%) were more likely to report lower levels of the eight developmental asset categories apart from Positive values and Constructive use of time, compared to those who did not (94%) (Table 3). Moreover, concerning the five environmental asset categories, participants who felt sad most or all the time and those who had attempted suicide before reported lower levels of the Personal, Social, Family, School, and Community assets relative to their respective counterparts who did not indicate that they had been sad most or all the time without cause or had attempted suicide (Table 3).

**Table 3:** Poor mental health indicators by mean number of assets experienced: *t*–test analysis.

**Asset Categories**	**Prolonged sadness**			**Suicide attempt**		
	**No *n* = 447**	**Yes *n* = 101**	**t**	**No *n* = 516**	**Yes *n* = 32**	**t**
The eight asset categories (range)						
Commitment to learning (0–7)	5.75	4.99	4.19^**^	5.66	4.75	3.01^**^
Positive values (0–7)	5.73	5.22	3.16^**^	5.64	5.50	0.51
Social competencies (0–7)	6.18	5.41	5.10^**^	6.06	5.53	2.08^*^
Positive identity (0–4)	3.04	1.39	11.55^**^	2.78	1.94	3.23^**^
Support (0–7)	5.08	3.97	5.99^**^	4.94	3.63	4.22^**^
Empowerment (0–6)	5.37	4.26	8.80^**^	5.23	4.19	4.73^**^
Boundaries & Expectations (0–9)	7.09	6.10	5.06^**^	6.96	5.78	3.58^**^
Constructive use of time (0–4)	1.80	1.62	1.60	1.78	1.53	1.38
The five environmental asset categories (range)						
Personal (0–11)	8.32	6.02	8.74^**^	7.95	6.91	2.25^*^
Social (0–12)	10.09	8.64	5.90^**^	9.90	8.34	3.76^**^
Family (0–9)	7.78	6.36	7.05^**^	7.60	6.00	4.66^**^
School (0–10)	8.60	7.46	5.23^**^	8.46	7.09	3.73^**^
Community (0–9)	5.27	4.48	4.41^**^	5.16	4.50	2.19^*^

* *p < 0.05*;

** *p < 0.01*.

In logistic regression analysis, when all the eight developmental asset categories were considered simultaneously in one model, only Positive identity and Empowerment were negatively related to prolonged sadness as expected, with odds ratios of *OR* = *0.50; 95% CI* = *0.41–0.61* and *OR* = *0.73; 95% CI* = *0.57–0.94*, respectively (Table 4A). Surprisingly, Constructive use of time was significantly and positively related to prolonged sadness, a finding that was not implied in the independent *t*–test analysis, and thus may be considered as a suppression effect. A suppression effect occurs when the direction of an association between two variables reverses after a third variable is introduced, and this could be due to a high correlation between the variables (Vatcheva et al., 2016). In model 2, when the demographic variables (i.e., age, gender, and parents' educational background) were controlled, only Positive identity remained significantly related to prolonged sadness, where a unit decrease in the asset category was associated with a 51% higher likelihood of being sad most or all the time *(OR* = *0.49; 95% CI* = *0.39–0.63)*, that is, when all other variables in the model were held at a constant. In Table 4B, using a significance level of 0.05, none of the eight asset categories was significantly related to the outcome, suicide attempt, in the two models (i.e., one with the eight asset categories alone, and the other, controlling for the demographic variables) that were analyzed.

**Table 4A:** Associations between the eight developmental asset categories and prolonged sadness: logistic regression analysis.

	**Model 1**					**Model 2**				
	***B***	***S.E*.**	***Sig***	***OR***	***95%CI***	***B***	***S.E*.**	***Sig***	***OR***	***95%CI***
Demographic variables										
Age						−0.26	0.17	0.128	0.77	0.55–1.08
Gender						0.29	0.33	0.376	0.134	0.70–2.57
Father's education						0.14	0.16	0.389	1.15	0.84–1.57
Mother's education						−0.11	0.20	0.592	0.90	0.60–1.33
Predictors										
Commitment to learn	0.06	0.09	0.475	1.06	0.09–1.26	0.02	0.11	0.825	1.02	0.83–1.26
Positive values	0.05	0.10	0.584	1.06	0.87–1.28	0.03	0.11	0.808	1.03	0.82–1.29
Social competencies	−0.04	0.11	0.698	0.96	0.78–1.19	−0.08	0.13	0.537	0.93	0.73–1.18
Positive identity	−0.69	0.10	***0.000***	0.50	0.41–0.61	−0.71	0.12	***0.000***	0.49	0.39–0.63
Support	−0.15	0.10	0.119	0.86	0.72–1.04	−0.13	0.12	0.251	0.88	0.70–1.10
Empowerment	−0.32	0.13	***0.015***	0.73	0.57–0.94	−0.19	0.16	0.216	0.82	0.61–1.12
Boundaries & Expectations	0.08	0.10	0.428	1.08	0.89–1.30	−0.00	0.11	0.969	1.00	0.80–1.24
Constructive use of time	0.30	0.14	*0.032*	1.35	1.03–1.79	0.41	0.17	*0.013*	1.51	1.09–2.09

*B, Unstandardized coefficient; S.E., Standard Error; Sig, Significance level; OR, Odds Ratio; CI, Confidence Interval*.

**Table 4B:** Associations between the eight developmental asset categories and suicide attempt: logistic regression analysis.

	**Model 1**					**Model 2**				
	***B***	***S.E*.**	***Sig***	***OR***	***95%CI***	***B***	***S.E*.**	***Sig***	***OR***	***95%CI***
Demographic variables										
Age						−0.23	0.26	0.363	0.79	0.48–1.31
Gender						−0.20	0.47	0.678	0.82	0.33–2.07
Father's education						0.00	0.24	0.710	1.09	0.68–1.76
Mother's education						−0.25	0.31	0.411	0.78	0.42–1.42
Predictors										
Commitment to learn	−0.09	0.12	0.452	0.92	0.73–1.15	0.01	0.15	0.959	1.01	0.75–1.36
Positive values	0.23	0.16	0.136	1.26	0.93–1.71	0.06	0.17	0.714	1.06	0.76–1.48
Social competencies	0.02	0.16	0.927	1.02	0.74–1.39	−0.03	0.18	0.849	0.97	0.69–1.37
Positive identity	−0.15	0.15	0.338	0.87	0.64–1.16	−0.23	0.18	0.195	0.79	0.56–1.13
Support	−0.23	0.14	0.109	0.80	0.61–1.05	−0.24	0.17	0.154	0.79	0.56–1.10
Empowerment	−0.27	0.19	0.154	0.77	0.53–1.11	−0.26	0.23	0.257	0.77	0.50–1.21
Boundaries & Expectations	−0.02	0.14	0.887	0.98	0.75–1.28	0.03	0.16	0.858	1.03	0.75–1.40
Constructive use of time	0.07	0.21	0.723	1.08	0.72–1.62	0.11	0.24	0.650	1.12	0.70–1.78

*B, Unstandardized coefficient; S.E., Standard Error; Sig, Significance level; OR, Odds Ratio; CI, Confidence Interval*.

For the assessment of the five environmental asset categories on prolonged sadness in logistic regression, Personal assets and Family assets were inversely related to prolonged sadness as expected, with odds ratios of *OR* = *0.72; 95% CI* = *0.64–0.82* and *OR* = *0.78; 95% CI* = *0.68–0.91*, respectively. The significant association remained for Personal assets, even after controlling for age, gender, and parents' educational background, while the influence of Family assets was no longer significant (Table 5A). When suicide attempt was analyzed as the outcome, and all the environmental asset categories were analyzed simultaneously, only the Family assets variable was significant, with an odds ratio of OR = 0*.80; 95% CI* = *0.65–0.98*. An odds ratio of *OR* = *0.79; 95% CI* = *0.63–1.01* when the demographic variables were controlled, showed that the influence of Family assets was now non–significant (Table 5B).

**Table 5A:** Associations between the five environmental asset categories and prolonged sadness: logistic regression analysis.

	**Model 1**					**Model 2**				
	***B***	***S.E*.**	***Sig***	***OR***	***95%CI***	***B***	***S.E*.**	***Sig***	***OR***	***95%CI***
Demographic variables										
Age						−0.32	0.16	0.050	0.73	0.53–1.00
Gender						0.62	0.31	0.043	1.86	1.02–3.37
Father's education						0.19	0.15	0.194	1.21	0.91–1.62
Mother's education						−0.03	0.19	0.887	0.97	0.68–1.40
Predictors										
Personal	−0.32	0.06	***0.000***	0.72	0.64–0.82	−0.29	0.07	***0.000***	0.75	0.65–0.86
Social	0.06	0.07	0.426	1.06	0.92–1.22	−0.02	0.09	0.794	0.98	0.82–1.16
Family	−0.24	0.08	***0.001***	0.78	0.68–0.91	−0.16	0.09	0.065	0.85	0.72–1.01
School	0.05	0.07	0.475	1.05	0.91–1.22	0.01	0.08	0.894	1.01	0.86–1.19
Community	0.04	0.09	0.672	1.04	0.87–1.24	0.00	0.11	0.997	1.00	0.81–1.23

*B, Unstandardized coefficient; S.E., Standard Error; Sig, Significance level; OR, Odds Ratio; CI, Confidence Interval*.

**Table 5B:** Associations between the five environmental asset categories and suicide attempt: logistic regression analysis.

	**Model 1**					**Model 2**				
	***B***	***S.E*.**	***Sig***	***OR***	***95%CI***	***B***	***S.E*.**	***Sig***	***OR***	***95%CI***
Demographic variables										
Age						−0.25	0.25	0.327	0.78	0.47–1.28
Gender						0.09	0.45	0.842	1.09	0.45–2.66
Father's education						0.16	0.24	0.489	1.18	0.74–1.87
Mother's education						−0.19	0.31	0.528	0.82	0.45–1.51
Predictors										
Personal	0.06	0.10	0.565	1.06	0.87–1.28	−0.06	0.11	0.615	0.95	0.76–1.17
Social	−0.10	0.11	0.392	0.91	0.73–1.13	−0.15	0.13	0.260	0.87	0.67–1.11
Family	−0.23	0.11	***0.032***	0.80	0.65 −0.98	−0.23	0.12	0.056	0.79	0.63–1.01
School	−0.08	0.11	0.446	0.92	0.75–1.14	0.01	0.12	0.925	1.01	0.79–1.29
Community	0.02	0.14	0.880	1.02	0.78–1.35	0.08	0.16	0.634	1.08	0.79–1.48

*B, Unstandardized coefficient; S.E., Standard Error; Sig, Significance level; OR, Odds Ratio; CI, Confidence Interval*.

## Discussion

The aim of the present study was to explore the role of Benson (2007) developmental asset categories on poor mental health indicators (prolonged sadness and suicide attempt) in Norwegian youth. In our study of the associations, we hypothesized negative associations between the two sets of factors, where higher scores on the assets will be associated with lower likelihood of prolonged sadness and suicide attempt. Findings from the independent *t*–tests indicated that higher levels of all eight developmental asset categories (i.e., Commitment to learning, Positive values, Social competencies, Positive identity, Support, Empowerment, Boundaries & Expectations, and Constructive use of time) except for Constructive use of time, were reported by youth who did not indicate prolonged sadness relative to their peers who did. However, only two of the asset categories (i.e., Positive identity and Empowerment) remained significant predictors of prolonged sadness when they were examined simultaneously in logistic regression; and Positive identity was the only significant predictor when demographic factors were also controlled. Similar findings were observed for the relations between the eight developmental asset categories and suicide attempt except for the logistic regression analyses, which evidenced no significant associations. For the five environmental asset categories (Personal, Social, Family, School and Community), findings from the independent *t*–tests indicated that youth who experienced more of these assets were also less likely to report prolonged sadness or attempted suicide, although in logistic regression only Personal assets and Family assets were significant predictors of prolonged sadness, and only Family assets predicted suicide attempt, when the asset categories were examined concurrently. However, when the demographic variables were also controlled the only significant association observed was between Personal assets and prolonged sadness.

Besides Constructive use of time, the report of the asset categories in the independent *t*–test was above average for our Norwegian sample, although youth without poor mental health reported more assets than those with a mental health problem. That youth who indicated poor mental health were also less likely to report the eight developmental and the five environmental asset categories was in line with the theoretical assumption of the Asset model (Benson et al., 2011). Accordingly, there is a cumulative effect of the assets where youth who report more assets are not only doing well on positive outcomes, but they are also protected against problems. Our findings confirmed this theoretical assumption as well as agreed with findings of earlier empirical studies of Pashak et al. (2018) along with Lenzi et al. (2015). Theoretically, it is posited that youth with protection through the experience of assets tend to engage in adaptive developmental regulations with their contexts, as their strengths (i.e., internal assets) optimally align with the resources and opportunities in their context (i.e., external assets). This developmental regulation is thought to be mutually beneficial. For youth who are not experiencing such protection through the experience of assets, one reason could be that they truly do not have access to such developmental assets. Alternatively, there could be a less than optimal alignment between their individual strengths and contextual resources resulting in the youth's inability to interact actively with the assets in their contexts and to be recognized as a contributor to themselves and others. When this happens, and the interaction is not beneficial to the youth or their context, a maladaptive developmental regulation can take place (Geldhof et al., 2019). However, it is important to note that findings from our independent *t*–tests only indicated a vertical pile–up of the assets (i.e., experiencing more assets in an asset category), while young people also need a horizontal stacking of the assets (i.e., experiencing assets across different asset categories) to effectively interact with their context.

In multivariate analysis of the role of the eight developmental assets, where a possible horizontal stacking of the assets was considered, only Positive identity and Empowerment emerged as significant predictors of prolonged sadness. Due to the positive correlations observed among the asset categories, it is possible that the other asset categories reinforced the influence of Positive identity and Empowerment. Nevertheless, the findings could indicate the importance of these two asset categories over the others. Earlier studies have often revealed the relative importance of internal assets over external assets for both positive youth outcomes (Adams et al., 2018) and negative development (Issa et al., 2020). In our study, Positive identity was in fact the only significant predictor of prolonged sadness, when demographic factors like age, gender and parents' educational background were also accounted for. Items measuring Positive identity were related to youth having control of their life and future, as well as feeling good about themselves and their future, among others. For young people, achieving a positive identity is a vital feat, which not only addresses who they currently are but as well, how they transition into healthy adulthood (Tsang et al., 2012; Ferrer–Wreder and Kroger, 2019).

In the assessment of the five environmental assets (before the adjustment of the demographics), Personal and Family assets emerged as significant predictors of prolonged sadness, while Family assets predicted suicide attempt. Like Positive identity, the emergence of Personal assets as a significant predictor is in line with earlier studies that highlight the importance of internal assets over external ones in terms of their prediction. The findings may also reflect the individualistic culture of the Norwegian context that encourages self–sufficiency in the population. However, before controlling for demographic factors, Family assets also emerged as a significant predictor of poor mental health and thus appeared to be protective against prolonged sadness and suicide attempt. From the perspective of Bronfenbrenner's ecological model (Bronfenbrenner, 2005), the influence of the immediate environment of young people like family can be more direct compared to distal environments, findings that were also observed earlier (Berry et al., 2018). Nevertheless, findings from the independent *t*–tests indicated that youth with no mental problems experienced more of the assets in all five environments compared to their peers who reported poor mental health, suggesting that resources and opportunities in both immediate and distal environments may be crucial to youth mental health. Indeed, the World Health Organization in their fight against poor mental health has emphasized the effect of community–level resources as well (World Health Organization, 2004).

### Limitations

Despite the important findings of the current study, there are some limitations that need to be mentioned. First, we used a convenience sample of high school students living in an urban district, which may not readily represent the youth or even the student population in Norway. Research with a more representative sample that includes youth within and outside the formal educational system as well as both rural and urban areas will aptly depict the developmental assets that are available to Norwegian youth. Again, as all constructs were measured by youth report, multiple perspectives and measurement approaches to developmental assets including environmental assets would be of added value. Moreover, a longitudinal layout in future studies rather than the cross–sectional design we used in our study (which cannot be used to provide information about causality) will enable researchers to have a better picture of the developmental effects of the assets on youth mental health and other youth outcomes.

The single items that were used in the measurement of prolonged sadness and suicide attempt were also not optimal; a more comprehensive measure of not only sadness, but of additional mental health indicators together with the developmental assets is needed in future studies to provide an accurate account of how the assets are truly protective of poor mental health in the Norwegian context.

Furthermore, whether the asset items accurately reflect available resources and opportunities in the Norwegian youth contexts remains to be addressed. For example, the internal consistency and youth report of the items that measured Constructive use of time was quite low (alpha of 0.44 for this scale in this sample) as many of them had experienced only a couple of these assets. The Constructive use of time asset category assessed among others, whether the youth participated in creative activities like music, theater, or other arts, and if they were involved in a church, mosque, or other religious group. These are indeed healthy arenas where the youth could develop skills and significant networks for positive development. However, many young people in Norway do not participate in religious activities, and involvement in recreational activities declines as they age. Thus, it appears that Constructive use of time for Norwegian youth are not being assessed well with these items. Moreover, even with those asset categories that registered higher scores, there is still the issue of whether assets unique to the Norwegian contexts were effectively captured by the items. All these questions need to be resolved in future studies using both quantitative and qualitative approaches to probe into the developmental assets of Norwegian youth as well as the ways in which they creatively and constructively spend their time.

### Implications for Research, Policy and Practice

Notwithstanding the limitations, the current findings have implications for research, policy and practice that seek to promote the development of diverse youth, including youth who experience poor mental health. For research, the findings of our study suggest that the Asset model can be used as a tool to explore the resources and opportunities that facilitate positive development and mental well–being among youth in Norway. By so doing, a critical assessment of the model can be undertaken to create a refined version that effectively depicts the quantities and qualities of the developmental assets of youth as well as assets within key environments. How the different assets reinforce each other in their role as protective or promotive factors can also be explored. Accordingly, efforts can be made to investigate assets in the model that are relevant for youth development but not readily available in the Norwegian context as well as assets unique to the Norwegian context. Such a refined and standardized model will be a useful tool in the study of positive development in diverse youth, not only in Norway, but in other Scandinavian contexts as well.

In terms of policy, the strategies outlined in Norway's youth policy together with other youth initiatives indicate that the country is doing quite well with respect to the resources and opportunities that are made available to youth. However, with its relatively high levels of poor mental health, more could be done to reduce barriers to accessing experiences, relationship and supports that promote developmental assets. In the making and modification of strategies outlined in youth policies and plans, attempts can be made to confirm that health and educational contexts that serve and interact with young people do not only focus on traditional medical ways of dealing with poor mental health but also arrange their activities in a way that will support easy access to an abundance of resources in the different contexts.

In terms of practice, our findings imply that mental well–being can be promoted if developmental assets are well–exploited, as youth that were showing indications of poor mental health were also reporting less resources and opportunities in almost all youth contexts. Thus, the PYD framework may be particularly well–suited to preventatively addressing mild to moderate forms of mental problems. While PYD advocates a universal intervention where all young people are targeted, it will not go against PYD principles to ensure that vulnerable groups, such as those experiencing poor mental health are engaging in adaptive and healthy interaction with their contexts. In their interaction with young people, youth contexts, such as the home, school and local community can implement strategies that ensure vertical pile–up (i.e., where youth experience more assets in an asset category) and horizontal stacking (i.e., where youth experience assets across different asset categories) along with an optimal, adaptive use of the developmental assets. While it appears from the current findings that internal or Personal assets are more influential, a collaborative effort between the youth contexts can also ensure that their respective resources and opportunities are being channeled effectively to meet the needs of all youth in their care.

## Conclusion

Theoretically, developmental assets represent resources and opportunities that tend to promote positive development as well as prevent risk and problem behaviors, along with poor health in young people. Empirical studies using mostly U.S. based samples but recently involving increasingly non–U.S. samples of youth have confirmed these associations. For risk and problem behaviors, developmental assets, such as social competence, family, and school support, as well as involvement in creative and community activities have been observed to protect against substance use, poor mental health, and other problem behaviors in young people (Lenzi et al., 2015; Lensch et al., 2018; Pashak et al., 2018). In the current study, less developmental assets were experienced by Norwegian youth who reported poor mental health (prolonged sadness and suicide attempt) than those who did not. Moreover, when the asset categories were examined together, Positive identity, Empowerment, Personal and Family assets appeared to be more important to protecting against poor mental health, especially prolonged sadness. However, internal and Personal assets turned out to be more important when demographic factors were considered. These are significant findings that can inform future research as well as youth policies and programmes in Norway. To effectively promote the development and mental well–being of youth, additional inquiry needs to be done with respect to how the Asset model is effectively capturing the personal and contextual resources and opportunities of diverse youth in Norway, along with how these assets are indeed making an impact on their mental health. This endeavor is important as the youth can subsequently transition into healthy adulthood and become active and important members of their community.

## Data Availability Statement

The raw data supporting the conclusions of this article will be made available by the authors, without undue reservation.

## Ethics Statement

The studies involving human participants were reviewed and approved by The Regional Committee for Medical and Health Research Ethics (REK), Norway. Written informed consent from the participants' legal guardian/next of kin was not required to participate in this study in accordance with the national legislation and the institutional requirements.

## Author Contributions

NW was responsible for data collection, conceptualization, original draft writing, methodology, software, formal analysis, and revisions. MB and LF-W contributed to conceptualization, methodology and revisions. All authors read and approved the version for publication.

## Conflict of Interest

The authors declare that the research was conducted in the absence of any commercial or financial relationships that could be construed as a potential conflict of interest.
